# Fixed-time artificial insemination in beef cattle

**DOI:** 10.1186/1751-0147-51-48

**Published:** 2009-11-30

**Authors:** Juhani Taponen

**Affiliations:** 1 Department of Clinical Veterinary Sciences, Faculty of Veterinary Medicine, University of Helsinki, Paroninkuja 20, 04920 Saarentaus, Finland

## Abstract

**Background:**

The study was designed to test the effect of fixed-time artificial insemination (fixed-AI) after the slightly modified Ovsynch protocol on the pregnancy rate in beef cattle in Finnish field conditions. The modification was aimed to optimize the number of offsprings per AI dose.

**Methods:**

Ninety Charolais cows and heifers were entered into the program an average of 1.8 times. Thus, 164 animal cases were included. Animals were administered 10-12 μg of buserelin. Seven days later animals without a corpus luteum (CL) were rejected (20.7%) while the remaining 130 cases with a CL were administered prostaglandin F_2α_, followed 48 h later with a second injection of buserelin (8-10 μg). Fixed-AI was performed 16-20 hours after the last injection.

**Results:**

The pregnancy rate was 51.5% (67/130). The pregnancy rate after a short interval (50-70 d) from calving to entering the program was significantly higher than that after a long interval (>70 d).

**Conclusion:**

This protocol seems to give acceptable pregnancy results in beef herds and its effect on saving labour is notable.

## Background

In breeding beef herds that produce genetically high merit breeding animals, it is necessary to use artificial insemination (AI) to import new genetic material from progeny tested bulls. Oestrus detection may, however, often be fairly problematic. Animals are usually kept in big herds and they are more difficult to approach and manage than dairy cattle. It may be difficult to identify a single animal from a big herd since in many beef breeds all animals are coloured similarly. Also, time spent with beef animals is much less than with dairy cattle. In some cases, catching a single animal for AI may be problematic. Because of these reasons, the use of AI after oestrus detection in some beef herds is either impossible or at least results in a poor pregnancy rate. According to Geary et al. [1], it is estimated that less than 5% of beef cows in the United States are artificially inseminated each year. Perhaps the biggest reason that so few beef cattle are artificially inseminated is the problem of accurate detection of oestrus.

Several hormonal treatments have been established to synchronise oestrus in order to facilitate and reduce time consumed on oestrus detection. Since the 1970s, when preparations of prostaglandin F_2α _were introduced into veterinary medicine [2-6], developing oestrus synchronisation programs has aroused great interest. During the first two decades, the AI after synchronisation programs was based, however, on oestrus detection, or alternatively, double inseminations on consecutive days [7-9]. In Finland, where the number of beef cattle is small, the semen used for breeding purposes has to be imported from abroad, and thus, it is fairly expensive. Owing to this, the programs demanding double inseminations are generally out of the question.

A protocol using gonadotropin-releasing hormone or its agonists (GnRH) and prostaglandin F_2α _or its agonists (PGF_2α_), called the Ovsynch protocol, was developed to synchronise ovulation in dairy cattle [10-14]. The objective of the Ovsynch protocol is to synchronise ovulation within an 8 hour period, enabling good fertility to fixed-time AI without oestrus detection [10]. The Ovsynch protocol has also been applied to postpartum beef cows [1]. The protocol consists of three hormonal treatments: the first one, GnRH, is intended to synchronise follicular waves, the second one, PGF_2α_, given 7 days later, induces luteolysis, and the third one, GnRH, given 36 to 48 hours after the PGF_2α _administration, induces ovulation at a pre-determined time. Artificial insemination is performed 16 to 24 hours after the second GnRH administration. Some variations exist in time periods between the treatments.

The Ovsynch protocol and its modifications are generally aimed to obtain the best possible conception rate with minimal need of labour. In circumstances explained above, one of the goals is to optimize the number of offspring per AI dose, since the target is to import new and valuable genetic material into the herd rather than conceive all animals per AI. In practice, the conception rate could be improved by selecting the involved animals according to the known facts that affect fertility. In addition, the selection method should be easy enough to apply in field conditions. Vasconcelos et al. [15] have shown that cows having low progesterone concentrations at the time of the administration of PGF_2α _in the Ovsynch protocol had significantly lower conception rate when compared to that of those cows having high progesterone concentration. The probability that included animals have high progesterone concentration at the time of the treatment can be increased in field conditions by detection of a corpus luteum by palpation of ovaries per rectum [16]. Instead Gümen et al. [17] have shown that anovulatory cows react well to Ovsynch protocol. Thus, on the day of the first GnRH injection of the Ovsynch protocol, there are not any clear criteria to select the cows into the program.

The aim of the study was to test the usefulness of fixed-time AI following the modified Ovsynch protocol, and its effect on the pregnancy rate in beef cattle managed in Finnish field conditions. The modification is aimed to optimize the number of offsprings per AI dose. In addition, the effect of season, parity, suckling and interval from calving to insemination on the pregnancy results are studied.

## Methods

The experiment was performed in a commercial Charolais elite herd having some 90 cows. In the herd, calvings are divided into two seasons, from March to April and from the end of October to the beginning of December. Thus, breeding periods are June - July and January - February. During the breeding periods the animals involved were loose housed in barns. The experiment lasted three years, from the summer of 1999 to the summer of 2002.

Altogether, seven breeding seasons, four in summer and three in winter, were included in the experiment. Before each season, 11 to 27 animals (mean 19) with a high breeding value were selected into the AI program. In all, 90 animals were entered into the program an average of 1.8 times (a maximum of 4 times for 5 animals). Thus, 164 animal cases were included in the experiment, of which 94 were on summer and 70 on winter seasons. Distributions of the animal cases into parity and interval between calving and entering the program classes are presented in Table 1. The majority of the cows were suckling; 19 cows did not lactate.

**Table 1:** Rejection rates (%) due to missing CL of animal cases in different parity * interval from calving to entering the program groups.

	Interval from calving			
	
	50-70	71-100	101-	Total
Parity				
0				27.3 (22)
1	25.0 (8)	10.7 (28)	25.0 (8)	15.9 (44)
2-5	18.2 (11)	16.9 (59)	25.0 (12)	18.3 (82)
6-	25.0 (4)	40.0 (10)	50.0 (2)	37.5 (16)
Total	21.7 (23)	17.5 (97)	27.3 (22)	

Numbers of animal cases in different groups are shown in parentheses.

In the beginning of the Ovsynch protocol, all animals selected to the program were administered 10 to 12 μg of buserelin (Receptal^R ^4 μg/ml, Intervet International B.V., Boxmeer, The Netherlands). Seven days later, the animals were manually examined per rectum by an experienced operator in order to determine the status of ovarian function. Animals not having a clear palpable corpus luteum (CL) were rejected from the program, while animals with a CL were administered 0.5 mg of cloprostenol (Estrumat^R ^0.25 mg/ml, Schering-Plough A/S, Ballerup, Denmark) or 0.15 mg of dexcloprostenol (Genestran^R ^0.075 mg/ml, Vetcare, Salo, Finland). Forty-eight hours after the treatment, the animals were administered 8 to 10 μg of buserelin. Fixed-time AI was performed 16 to 20 hours after the last injection with semen of bulls of known normal fertility.

Eighteen days after the AI, a bull was introduced to the herd in order to serve cows that returned to oestrus. Pregnancy examinations were performed by palpation per rectum six to eight weeks after the AI, when it was easy to detect pregnancies conducted from AI. All examinations were made by one and the same experienced veterinarian.

Data were analysed using the SPSS 13.0 for Windows software. To study the effect of season, suckling, parity and interval between calving and entering the program among cows on the rejections and pregnancy rates, the data were analysed using logistic regression for binomially distributed data. Before analyses, parity was classified to three groups, first parity cows, middle-aged cows (parity 2-5) and old cows (parity 6-), and interval between calving and entering the program to three groups, short (50-70 days), medium (71-100 days) and long (101-days) interval. For parity and interval, the last and first category, respectively, served as a reference category. Initially, all the variables were included in the analysis. In the backward stepwise analysis procedure, non-significant variables were omitted. Chi-square test was used to analyse the differences in rejections and pregnancy rates between cows and heifers. P-values less than 0.05 were considered significant.

## Results

Of 164 animal cases, 34 (20.7%) were rejected from the program since no CL were detected on the day of cloprostenol/dexcloprostenol administration. During summer and winter seasons, 20.2% and 21.4% were rejected, respectively. The rejection rates in parity * interval between calving and entering the program (later "time interval") groups are presented in Table 1. Differences in rejections in cows between parity (p = 0.132), suckling (p = 0.160) and time interval groups (p = 0.214) and seasons (p = 0.932) were not statistically significant. The rejections in heifers were not significantly different (p = 0.416) from those in cows. However, heifers and old cows were rejected numerically 10 to 20 percentage points more than younger, parity 1-5, cows.

During the experiment 130 AIs were performed, of which 67 (51.5%) led to pregnancy. Distributions of the AIs into parity and time interval classes are presented in Table 2. In summer and winter seasons, the pregnancy rates were 53.3% and 49.1%, respectively. Effects of season and suckling were not statistically significant (p = 0.964 and p = 0.776, respectively). Thus, these variables were omitted form the final analysis. The pregnancy rates in parity * time interval groups are presented in Table 2. Time interval from calving to the start of the program affected conception. The differences in pregnancy rates in cows between the three time interval groups were statistically significant (p = 0.029). In particular, pregnancy rate in the group having medium time interval (71-100 days, p = 0.019) as well as in the group having long time interval (100-days, p = 0.010) were significantly lower when compared to the reference group with short interval (50-70 days).

**Table 2:** Pregnancy rates (%) after fixed-time artificial insemination in different parity * interval from calving to entering the program groups.

	Interval from calving			
	
	50-70	71-100	101-	Total
Parity				
0				43.8 (16)
1	83.3 (6)	56.0 (25)	16.7 (6)	54.1 (37)
2-5	100.0 (9)	49.0 (49)	55.6.(9)	56.7 (67)
6-	0.0 (3)	33.3 (6)	0.0 (1)	20.0 (10)
Total	77.7 (18)	50.0 (80)	37.5 (16)	

Numbers of artificial inseminations in different groups are shown in parentheses.

The effect of parity on pregnancy rate was not as clear as the effect of time interval. The overall differences between the three parity groups of cows tended to be statistically significant (p = 0.063). However, the pregnancy rates of the first parity and middle-aged cows, when contrasted to that of the old cows (the reference group), were significantly (p = 0.032 and p = 0.019, respectively) higher. The pregnancy rates in heifers were not significantly different (p = 0.506) from those in cows. However, the pregnancy rates in heifers seemed to be numerically somewhat lower than in parity 1-5 cows.

## Discussion

The average pregnancy rate of 51.5% was reached with a slightly modified Ovsynch protocol in this Charolais beef herd. During the preceding years, the approximate pregnancy rate after AI based on oestrus detection had varied around 20% when calculated according to the herd bookkeeping. No data about overall pregnancy results after AI in beef cattle in Finland are available, but in dairy cattle, the 60-days non-return rate is about 63%, and thus, the real pregnancy rate can be expected to be very close to 50%.

Originally the Ovsynch protocol for synchronisation of ovulation was developed for reproductive management in dairy herds [10]. Since this paper, many studies have evaluated the fertility of lactating dairy cows following the Ovsynch protocol, and pregnancy rates per AI have varied from 27% to 39% [11-13,15,18,19]. These pregnancy rates have been similar to [11,12] or only slightly lower [18,20] than the pregnancy rates with AI after oestrus detection or after oestrus detection following synchronisation of oestrus with PGF_2α _in the control cows. The Ovsynch protocol has also been applied for beef cattle. Geary et al. [21] have reported a pregnancy rate of 52% in beef cows after the Ovsynch protocol. In the same study, they also modified the Ovsynch protocol by adding 48-h calf removal from the PGF_2α _administration to the second GnRH treatment. This calf removal tended to increase the pregnancy rate (from 52 to 61%). Geary et al. [1] earlier carried out a quite similar experiment where the Ovsynch protocol with 48-h calf removal led to a pregnancy rate of 54%, suggesting that the pregnancy rate without calf removal would be slightly below 50%. Two other studies have obtained similar results; pregnancy rates have varied from 47.7% to 53% after the Ovsynch protocol in beef cows [22,23]. These results are quite identical and accord well with the results of the present study.

Several modifications for the Ovsynch protocol have been developed. For example a strategy known as Cosynch eliminates one cow handling period (the second GnRH and AI at the same time) and facilitates once-daily restraint of cows for administration of hormone injections and timed AI. In Cosynch protocols, the time interval between the PGF_2α _administration and the second GnRH + AI has varied from 48 h to 72 h. Pregnancy rates after the use of the Cosynch method have been similar or slightly lower than those obtained in the Ovsynch [19,24]. Despite this the Cosynch method should be remembered as an alternative in beef herds, where the handling facilities may be poor. The fact that the conception rate is affected by the stage of the oestrous cycle at the beginning of the protocol [15] has led to the development of presynchronisation methods preceding the Ovsynch and Cosynch protocols. The presynchronisation methods include either two PGF_2α _administrations before the first GnRH injection (Presynch) [25] or a progesterone releasing intravaginal device inserted for the first seven days of the program [26]. In beef cattle, presynchronisation with progesterone supplementation has been studied, but no additive effects were detected [27]. It should be kept in mind that all these handlings and treatments cause extra costs, and it is questionable whether these are cost-effective.

Interestingly, the fertility after fixed-time AIs seems to be better following shorter rather than longer intervals from parturition to start of the protocol. The low sample sizes among some of the cells (Table 2) limit the effective analysis for interactions, but observed percentages of pregnancy rates suggest that long interval from calving to the start of the program may be a risk factor, especially for the first parity cows. This finding disagrees with the well known fact obtained in dairy cattle that the conception rate to AI increases along an increasing interval from parturition to at least 120 days [28,29]. Nevertheless, in line with our finding, Geary et al. [1] have obtained similar results in beef cows after the Ovsynch protocol. The pregnancy rates were 74%, 51% and 52% when the cows entered the program <70, 70-90, and >90 days post partum, respectively. The fairly poor pregnancy results in the groups having a long interval from parturition to start of the protocol can partly be explained with a bias in the selection of animals into the groups. It is possible that the group of animals with a long interval might have included more animals that had had problems in reproductive functions. However, even a more interesting finding is that fertility was best in those animals having the shortest interval from the calving. In dairy cattle, fertility during the corresponding period is lowest, and a negative energy balance during the first weeks after the calving has been shown to be one of the most important reasons for the poor fertility [30,31]. In suckling beef cattle, the milk yield is clearly lower, and thus negative energy balance flatter than in dairy cattle, which could be one explanation for the excellent fertility fairly soon after calving. However, in beef cattle, suckling has been shown to be one of the major factors in determining the length of puerperal anoestrus [32]. In our study, this did not seem to affect the fertility after 50 days after calving.

The synchronisation program used in this study was slightly modified from the original Ovsynch protocol which principally does not include any clinical examinations of the animals entering the program. In the present study, ovaries of the involved animals were palpated per rectum, and those animals not having a functional CL were omitted from the program. One of the goals of the program was to optimize the number of offspring per AI dose, since the target was to import new and valuable genetic material into the herd rather than conceive all animals per AI. Vasconcelos et al. [15] have reported that of cows having low progesterone concentrations at the time of the administration of PGF_2α_, 68% of them had a synchronized ovulation compared to an overall synchronization rate of 87% in all cows - keeping in mind that these cows were cyclic. In addition to the fact that cyclic cows having low progesterone concentrations at the time of the administration of PGF_2α _had a distinctly lower synchronisation rate, the pregnancy rates in anoestrous cows have been lower than in cyclic cows (48% vs. 62%) [21]. Similar, although not significant, differences have been reported by Geary et al. [1].

The Ovsynch regimen does not seem to be as effective for synchronizing heifers as lactating dairy cows. Statistically, there was a lower percentage of heifers that responded to the first injection of GnRH, and this may have resulted in only 75% of heifers being synchronized compared to 100% of cows [10]. According to Pursley et al. [12], heifers have a lower pregnancy rate per AI after Ovsynch than after PGF_2α _and detected oestrus. They concluded that Ovsynch may be the first synchronisation protocol that has performed well in lactating dairy cows but not in heifers. Although the number of heifers involved in the present study was low, and thus, significant results were not reached, it seemed that proportionally more heifers were rejected from the program due to unresponsiveness; the conception rate was 7.7 percentage points lower than the overall average.

## Conclusion

The use of fixed-time AI after the Ovsynch protocol in beef herds seems to give acceptable pregnancy results, and its effect on the saving of labour is notable. However, final profitability depends much on the herd and the objectives involved. Season and suckling during the insemination period did not seem to affect fertility. Interval from calving to start of the protocol had a clear influence: the fertility was the best in the group having the shortest interval, 50-70 days. The effect of parity was not clear, but it seemed that this protocol does not lend for heifers as well as for cows.

## Competing interests

The author declares that he has no competing interests.
